# Are young men attempting to achieve paternity through intracytoplasmic sperm injection candidates for general health screenings?

**DOI:** 10.1038/s41443-024-01009-y

**Published:** 2024-12-06

**Authors:** Michael Kitlinski, Aleksander Giwercman, Yvonne Lundberg Giwercman, Angel Elenkov

**Affiliations:** 1 Department of Translational Medicine, Lund University, Malmö, Sweden; 2 Reproductive Medicine Centre, Skåne University Hospital, Malmö, Sweden

**Keywords:** Preventive medicine, Reproductive disorders

It is now well established that men from infertile couples are at increased risk of a number of non-communicable diseases, both malignant and non-malignant [1]. These findings call for reconsideration of the criteria for what can be considered as good health service when meeting men with fertility problems. Thus, it has been discussed if it is sufficient to only help these men to become fathers or if prevention of long-term morbidity and mortality also should be considered as standard care. Such routines in the management of men from infertile couples are currently not recommended, although the clinical guidelines from the European Association of Urology mention discussing general health status with men having fertility issues [2]. One of the major obstacles in this regard is to define the subgroups of infertile men in whom such actions would be most feasible and cost-efficient.

The recently published article by Saffati et al. dives deep into the topic of male infertility and its long-term health consequences, where it provides valuable insight on how poor sperm quality as a proxy for general health could be implemented as a screening tool for young men in the future [3]. While one could argue that reproductive ability as a screening tool for certain diseases in young men is worth to consider as a part of standard medical care, semen analysis as a screening tool has its downfalls.

Standard semen parameters are subject to significant intra-individual day-to-day variation [4, 5], and therefore a single semen analysis may not be sufficient to identify high-risk subjects. Furthermore, not included in standard semen routine analyses, other sperm characteristics affecting fertility could also be impaired [6], as for example DNA Fragmentation Index (DFI). It has been shown that up to 25% of men from infertile couples with otherwise normal semen parameters, have an increased level of sperm DFI [7]. High DFI is not only associated with decreased fertility, but also with impaired insulin sensitivity [8].

An alternative would be to focus on other proxies for male fertility potential, as for example men attempting to achieve paternity through intracytoplasmic sperm injection (ICSI). For men with very few sperm, or spermatozoa with poor function, the only option for paternity is ICSI treatment, in which a sperm is injected into an egg and the embryo put back into the uterus. Targeting this group of men could have several advantages as in many countries, ICSI treatments are mostly limited to couples with so called male factor infertility. Thus, being referred for ICSI could serve as a proxy for significant deviations in sperm quality and/or quantity under sufficiently long period to have a clinical implication, namely infertility. As degree of impaired reproductive function is known to be associated with the risk of future adverse health outcomes [9, 10], men with non-obstructive azoospermia might be the subgroup of ICSI-men which might benefit the most from somatic health examinations. Furthermore, men trying to achieve paternity through ICSI represent a quite significant proportion of the population and are already in contact with the health care system making them an easily accessible target group for screening and preventive measures.

Implementing basal routine examinations or screening tests for men from couples qualified for ICSI, could be performed simultaneously or shortly after the fertility evaluation. Such implementations might not only be cost-effective, but also provide logistical advantages. By introducing basal health investigations in routine clinical work-up for couples undergoing fertility care, it would result in earlier detections of diseases in young men where preventive measures would decrease the number of future hospital admissions and healthcare costs if the disease was detected and/or treated before it became evident due to significant clinical symptoms. Furthermore, as these men already are willing to achieve paternity with help from the health care system, a screening would likely not imply any additional stigma due to additional basal health examinations. Spectrum of general health investigations which should be considered in males trying to achieve paternity through ICSI, should include blood-pressure measurements [11], anthropometric measurements (body-mass-index, waist circumference) [12], serum levels of reproductive hormones (testosterone, luteinizing hormone, follicle stimulating hormone, sex hormone binding globulin) [13] and simple tests to discover metabolic disturbances (e.g., HOMA_IR_ or TyG index) [14]. Furthermore, scrotal ultrasound to exclude non-palpable testicular tumors [15] or measurement of Prostate Specific Antigen levels to detect prostate cancer [16], could also be beneficial for subfertile men. It remains however to be proven whether introducing the above-mentioned general health examinations in standard fertility care, will cost-effectively decrease infertility-related morbidity or mortality in men seeking ICSI treatment.

On the other hand, in countries with limited access to assisted reproduction treatment due to socio-economic factors, alternative strategies for such early screening need to be developed. In some parts of the world, the indications for using ICSI in infertile couples have now been widened to also include cases of unexplained fertility, advanced maternal age, low oocyte yield or poor quality of the latter. Therefore, attempts on targeted screening campaigns, as suggested by us, should be done in the setting where ICSI referral is indicated by male-factor infertility [17] only, and preferably in countries with tax-funded assisted reproduction.

Although simple semen analysis could be used as a proactive health measure, we believe targeting infertile men seeking to achieve paternity by ICSI to be more feasible and cost-effective in preventing infertility-related morbidity, a concept which, in our earlier publications, was shown to be well-grounded [18, 19]. Nevertheless, whether it is poor sperm quality based on basic semen analysis or attempted paternity through ICSI, reproductive ability as a marker for poor somatic health in young men may in the future have promising clinical implications. Implementation of targeted health interventions based on a male’s fertility potential grows even more so relevant as we each day keep learning about the negative implications of impaired testicular function beyond its reproductive goals.
